# Cellular Management of Zinc in Group B Streptococcus Supports Bacterial Resistance against Metal Intoxication and Promotes Disseminated Infection

**DOI:** 10.1128/mSphere.00105-21

**Published:** 2021-05-19

**Authors:** Matthew J. Sullivan, Kelvin G. K. Goh, Glen C. Ulett

**Affiliations:** a School of Pharmacy and Medical Sciences, Griffith University, Gold Coast, Australia; b Menzies Health Institute Queensland, Griffith University, Gold Coast, Australia; University of Arizona

**Keywords:** *Streptococcus agalactiae*, bacterial pathogenesis, *czcD*, metal ions, metallobiology, zinc efflux

## Abstract

Zinc is an essential trace element for normal bacterial physiology but, divergently, can intoxicate bacteria at high concentrations. Here, we define the molecular systems for Zn detoxification in Streptococcus agalactiae, also known as group B streptococcus, and examine the effects of resistance to Zn stress on virulence. We compared the growth of wild-type bacteria and mutants deleted for the Zn exporter, *czcD*, and the response regulator, *sczA*, using Zn-stress conditions *in vitro*. Macrophage antibiotic protection assays and a mouse model of disseminated infection were used to assess virulence. Global bacterial transcriptional responses to Zn stress were defined by RNA sequencing and quantitative reverse transcription-PCR. *czcD* and *sczA* enabled S. agalactiae to survive Zn stress, with the putative CzcD efflux system activated by SczA. Additional genes activated in response to Zn stress encompassed divalent cation transporters that contribute to regulation of Mn and Fe homeostasis. *In vivo*, the *czcD*-*sczA* Zn management axis supported virulence in the blood, heart, liver, and bladder. Additionally, several genes not previously linked to Zn stress in any bacterium, including, most notably, *arcA* for arginine deamination, also mediated resistance to Zn stress, representing a novel molecular mechanism of bacterial resistance to metal intoxication. Taken together, these findings show that S. agalactiae responds to Zn stress by *sczA* regulation of *czcD*, with additional novel mechanisms of resistance supported by *arcA*, encoding arginine deaminase. Cellular management of Zn stress in S. agalactiae supports virulence by facilitating bacterial survival in the host during systemic infection.

**IMPORTANCE** Streptococcus agalactiae, also known as group B streptococcus, is an opportunistic pathogen that causes various diseases in humans and animals. This bacterium has genetic systems that enable zinc detoxification in environments of metal stress, but these systems remain largely undefined. Using a combination of genomic, genetic, and cellular assays, we show that this pathogen controls Zn export through CzcD to manage Zn stress and utilizes a system of arginine deamination never previously linked to metal stress responses in bacteria to survive metal intoxication. We show that these systems are crucial for survival of S. agalactiae *in vitro* during Zn stress and also enhance virulence during systemic infection in mice. These discoveries establish new molecular mechanisms of resistance to metal intoxication in bacteria; we suggest these mechanisms operate in other bacteria as a way to sustain microbial survival under conditions of metal stress, including in host environments.

## INTRODUCTION

Inside living cells, zinc is an essential cofactor for metalloenzymes (1, 2) but is toxic at high concentrations, as can be encountered by bacteria inside phagocytes (3, 4). The double-edged sword of essentiality and toxicity of Zn to bacteria is a burgeoning area of research due to the potential for antimicrobial applications (5–7). In the host, bacterial pathogens employ distinct mechanisms for internalizing essential Zn (8, 9), and, in turn, the host can restrict Zn availability as an antimicrobial strategy (10). Phagocytes can mobilize cellular Zn to expose internalized bacteria to metal concentrations that are antimicrobial (5, 11, 12). Host-driven Zn intoxication (defined as an excess of extracellular Zn) of bacterial pathogens can involve ablation of uptake of essential Mn (13), a compromised bacterial response to oxidative stress (14), or disrupted central carbon metabolism (15). Some bacteria can evade metal intoxication by mechanisms that involve metal efflux (16).

In streptococci, a specific genetic system manages Zn homeostasis by regulating metal import and export (13, 17). In pneumococcus and Streptococcus pyogenes, systems for Zn efflux pair a Zn-sensing transcriptional response regulator (*sczA*) with a Zn efflux transporter, encoded by *czcD* (17, 18). Here, we studied Zn management in Streptococcus agalactiae, also known as group B streptococcus, which is an important opportunistic pathogen that has undefined Zn detoxification systems and is associated with distinct disease etiologies compared to other streptococci. We establish a role for CzcD, as well as other novel factors, in mediating S. agalactiae resistance to Zn stress and virulence.

## RESULTS

### Excess Zn impedes S. agalactiae growth and disturbs cell physiology.

**FIG 1:**
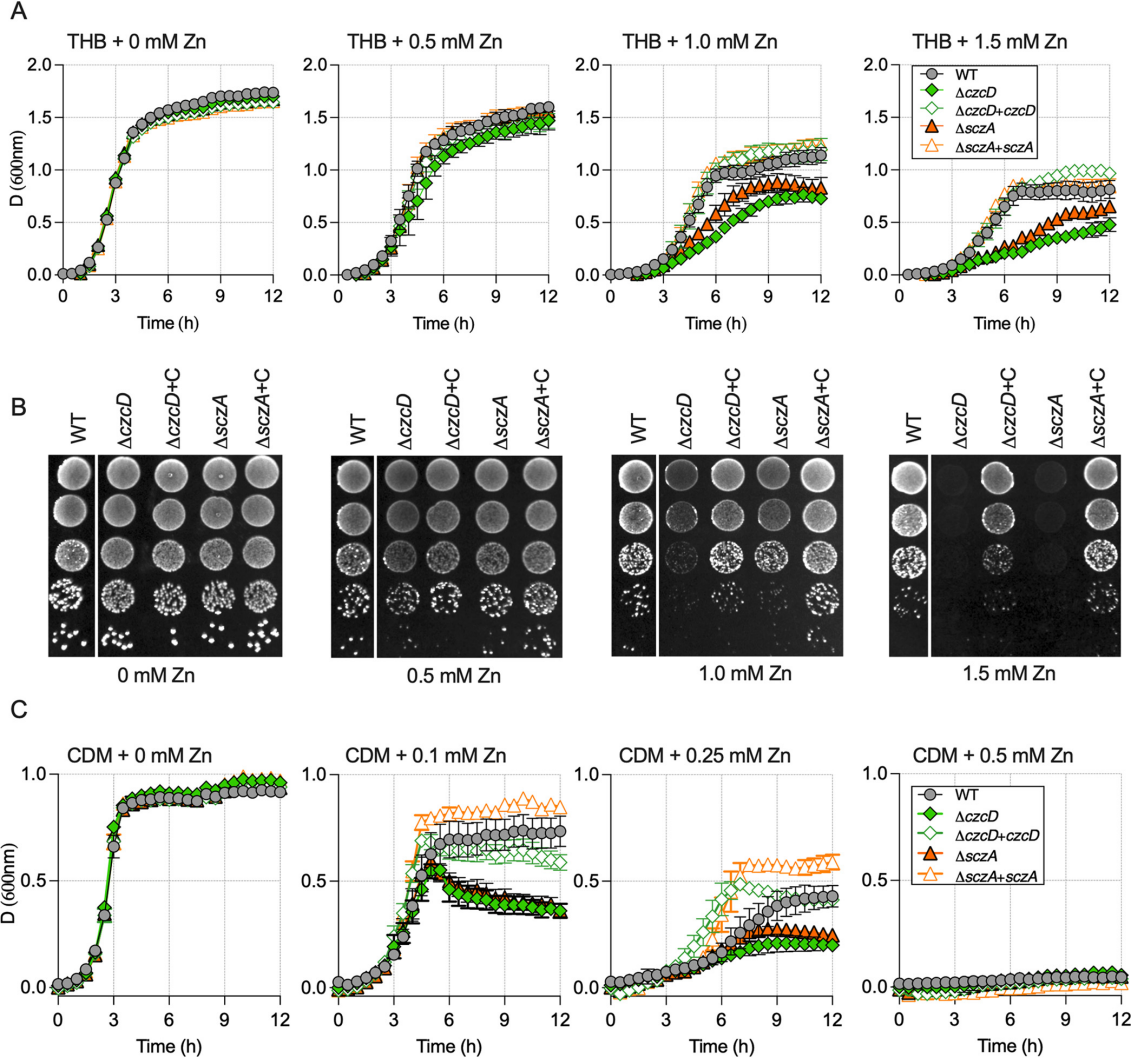
Comparison of the growth of WT S. agalactiae, Δ*czcD* and Δ*sczA* isogenic mutants, and complemented strains. (A) Growth curves of 874391 and mutant strains, as indicated, in Todd-Hewitt broth (THB) supplemented with 0, 0.5, 1.0, and 1.5 mM Zn. (B) Analysis of growth of S. agalactiae strains on solid TH medium with increasing Zn concentrations by serial dilution and droplets on agar. Composite images were spliced for labeling purposes. All five strains were imaged from the same agar plate for each Zn concentration. (C) Growth curves of 874391 and mutant strains, as indicated, in CDM supplemented with 0, 0.1, 0.25, and 0.5 mM Zn. Data shown are mean measurements of attenuance (*D*; at 600 nm) from 3 to 4 independent experiments, and bars show standard errors of the means (SEM).

Initial assays analyzed the growth of wild-type (WT) S. agalactiae strain 874391 (ST-17; serotype III) in a nutrient-rich Todd-Hewitt broth (THB) supplemented with moderate (0.5 mM), high (1.0 mM), and excess (1.5 mM) levels of Zn. High and excess Zn (≥1 mM) delayed exponential growth of the WT, with significant attenuation of the growth rate and final biomass yield (Fig. 1A). An isogenic Δ*czcD* mutant strain was significantly more susceptible to Zn at ≥1 mM (Fig. 1A). Full-length *czcD* supplied in *trans* to the mutant (Δ*czcD*+*czcD*) restored growth to WT levels in Zn stress (Fig. 1A). Spot test assays of the bacteria on agar containing 0, 0.5, 1.0, and 1.5 mM Zn showed similar levels of susceptibility of the WT and Δ*czcD* strains on solid medium (Fig. 1B) compared to planktonic growth.

We next used a nutrient-limited medium to examine Zn stress in S. agalactiae in a modified chemically defined minimal medium (CDM), which likely more closely reflects a host environment (nutrient limited). CDM contained low basal levels of Zn (0.11 ± 0.03 μM), as determined by inductively coupled plasma optical emission spectroscopy (ICP-OES). Growth assays of S. agalactiae in CDM with or without Zn supplementation revealed markedly enhanced Zn-induced toxicity compared to THB, with Zn being totally bacteriostatic to all strains at 0.5 mM (Fig. 1C). Similar to THB, growth of the Δ*czcD* mutant was significantly inhibited compared to that of the WT upon exposure to ≥0.1 mM in CDM (Fig. 1C).

### Regulation of Zn efflux in S. agalactiae.

**FIG 2:**
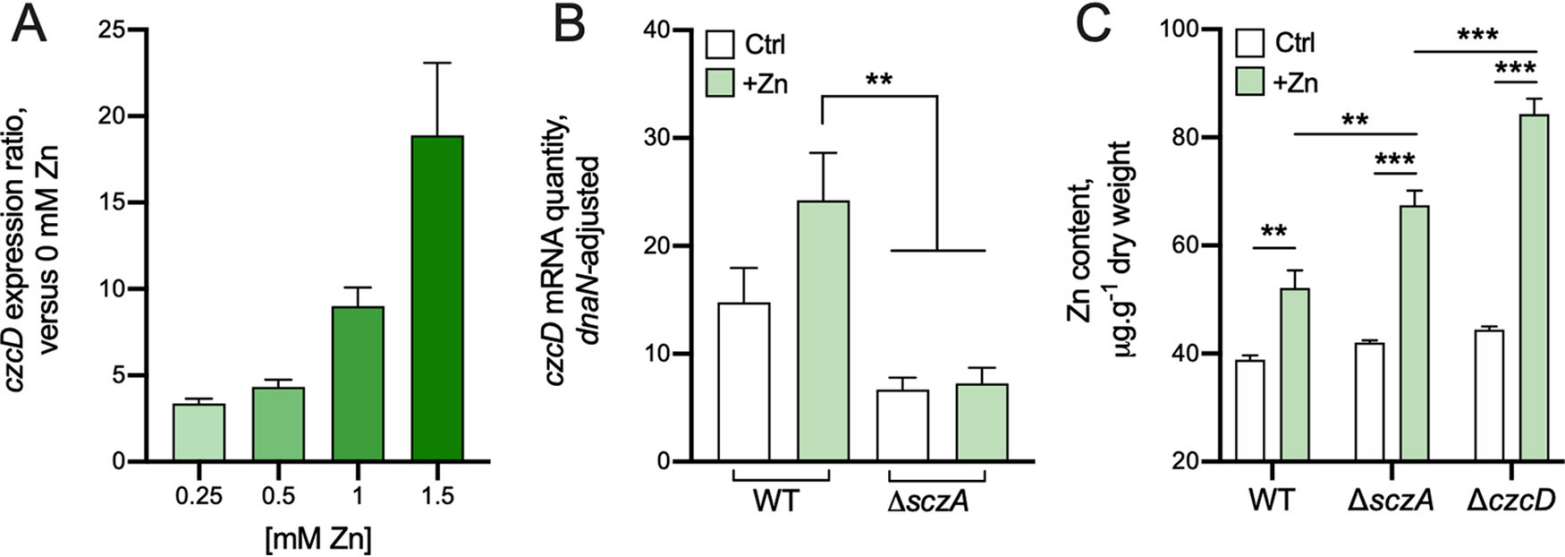
Expression analysis of *czcD* and intracellular Zn content in *S. agalactiae* strains. (A) Expression ratio of *czcD* quantified by qRT-PCR in THB medium containing 0.25, 0.5, 1.0, and 1.5 mM Zn compared to THB without Zn. (B) Relative *czcD* transcripts were quantified in WT and Δ*sczA* strains with and without Zn supplementation. (C) Intracellular accumulation of Zn was compared with and without Zn supplementation in WT, Δ*sczA*, and Δ*czcD* strains. Ratios in panel A were calculated, as described previously (45), using *C_T_* values, primer efficiencies, and housekeeping *dnaN*. In panels B and C, Ctrl, THB + 0 mM; +Zn, THB + 0.25 mM Zn. Bars show means and SEM from 3 to 4 independent experiments and compared by one-way analysis of variance (ANOVA) with Holm-Sidak multiple comparisons (**, *P* < 0.01; ***, *P* < 0.001).

The capacity of Zn stress to induce expression of *czcD* for Zn export was examined by analyzing mid-log-phase S. agalactiae grown with 0.25 to 1.5 mM supplemental Zn for 2.5 h prior to quantitative reverse transcription-PCR (qRT-PCR) quantification of *czcD*. *czcD* was significantly upregulated in response to Zn (3-fold to 18.9-fold) in a manner that was titratable with the Zn concentration (Fig. 2A) and consistent with a role for CzcD in responding to extracellular Zn. Interestingly, we identified a candidate gene, divergent from *czcD*, that encodes a putative Zn-responsive activator in the TetR family of transcription factors, termed streptococcal *czcD* activator, or *szcA* (19). Similar to Δ*czcD* S. agalactiae, a mutant deficient in *sczA* (Δ*sczA*) was markedly more susceptible to Zn stress than the WT under both nutrient-rich and -limited conditions; the attenuation was restored by complementation *in trans* (Fig. 1).

To confirm the role of SczA as a Zn-responsive activator of *czcD* expression, we quantified *czcD* mRNA in Δ*sczA* S. agalactiae grown in 0.25 mM supplemental Zn (subinhibitory; to enable comparisons independent of metabolic state and alleviate potential bias from any discordant Zn stress between WT and mutants with varied resistance phenotypes). Deletion of *sczA* significantly perturbed activation of *czcD* in response to Zn (Fig. 2B), consistent with previous reports of SczA functioning as a Zn-dependent activator of *czcD* in S. pyogenes and S. pneumoniae (17, 18).

### Intracellular Zn content in S. agalactiae during Zn stress.

We analyzed accumulation of Zn ions within the bacteria following Zn stress by using growth conditions equivalent to those for transcriptional experiments. WT, Δ*czcD*, and Δ*sczA* S. agalactiae were grown in 0.25 mM supplemental Zn for 2.5 h prior to quantifying intracellular metal content by ICP-OES. In the WT strain, Zn stress caused accumulation of intracellular Zn (52.1 versus 38.8 μg Zn/g dry weight) compared to control cultures without supplemental Zn. In addition, intracellular Zn contents in Δ*czcD* and Δ*sczA* mutants grown under equivalent conditions were significantly enhanced (84.3 and 67.5 μg Zn/g dry weight, respectively) compared to the WT in Zn stress or control incubations of the mutant strains without Zn (Fig. 2C). These findings are consistent with roles for CzcD and SczA as mediators of Zn efflux.

### Resistance of intracellular S. agalactiae to Zn stress in macrophages.

**FIG 3:**
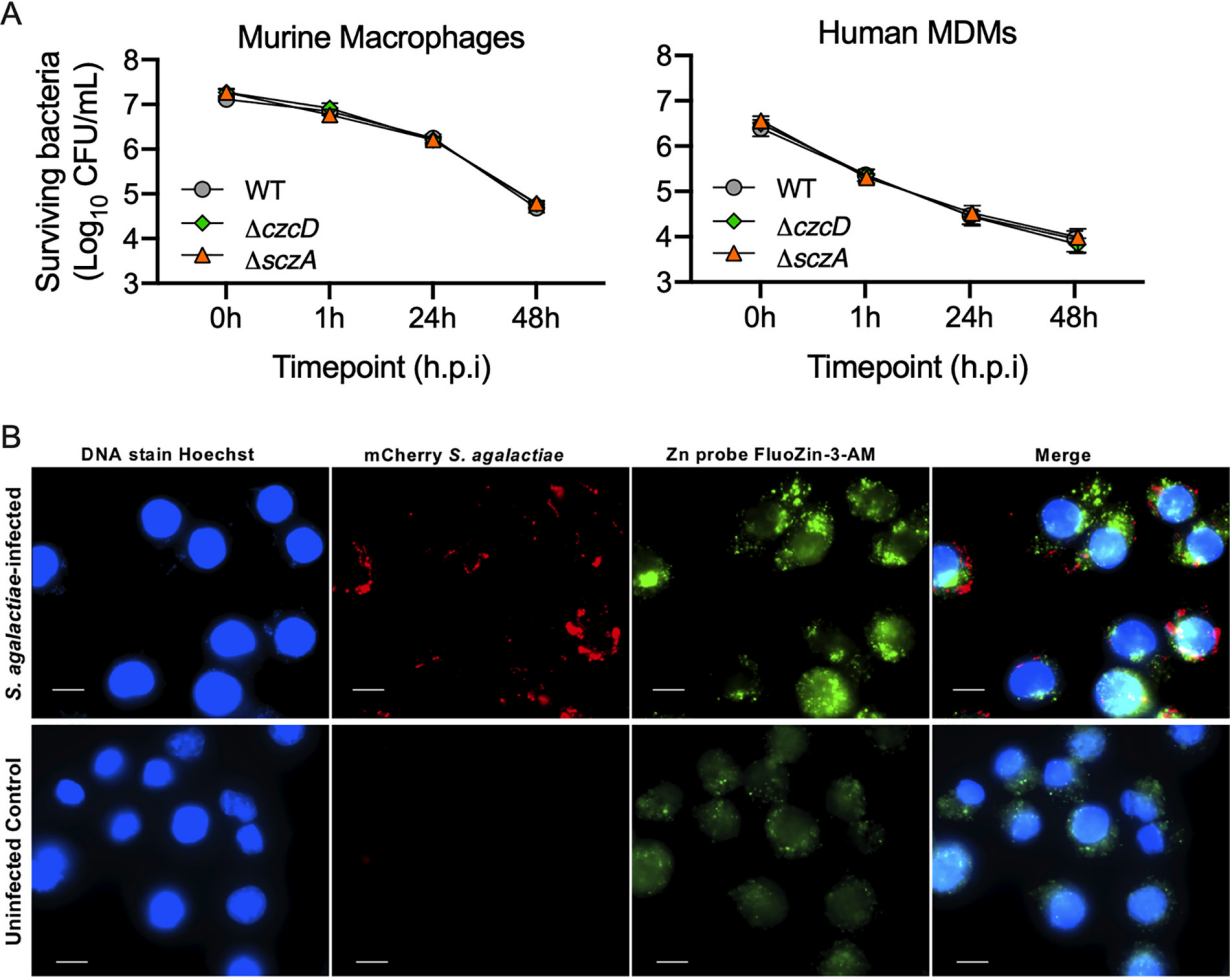
Interactions of S. agalactiae with macrophages and consequential mobilization of Zn. (A) Antibiotic protection assays with WT, Δ*sczA*, and Δ*czcD* strains and mouse (J774A.1) or human (U937 monocyte-derived macrophage-like) macrophages, with surviving bacteria quantified at 0, 1, 24, and 48 h postinfection (h.p.i.). Data are means and SEM from 4 to 5 independent experiments. (B) Fluorescence imaging of the release of free Zn (detected using FluoZin-3 AM) by J774A.1 cells infected with WT S. agalactiae expressing mCherry from pGU2665 compared to noninfected J774A.1 cells at 24 h postinoculation. DNA was stained using Hoechst 33258. Scale bars, 10 μm.

To determine if Zn efflux systems support survival of S. agalactiae in phagocytic cells, we used murine macrophages and human monocyte-derived macrophage-like cells in antibiotic protection assays at 1 h, 24 h, and 48 h of incubation. Viable intracellular S. agalactiae cells were reduced in number over the time course in human and murine cells, but no significant differences between WT, Δ*czcD*, and Δ*sczA* strains were detected (Fig. 3A). To examine host-mediated Zn release inside phagocytic cells, we developed a system for expressing mCherry in S. agalactiae. We used this in concert with a Zn-binding fluorophore (FluoZin-3AM) to visualize intracellular S. agalactiae inside J774A.1 cells. Despite no effect of Zn efflux mutants on survival, we observed that S. agalactiae induced robust mobilization of Zn inside host cells at the 24-h time point compared to noninfected control incubations (Fig. 3B), as shown by enhanced detection and distribution of free Zn using FluoZin-3AM.

### S. agalactiae Zn efflux systems contribute to virulence *in vivo*.

**FIG 4:**
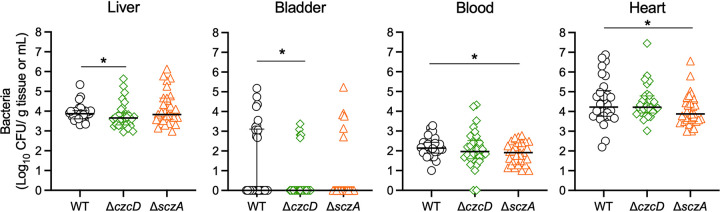
Virulence of WT (gray circles), Δ*czcD* (green diamonds), and Δ*sczA* S. agalactiae (orange triangles) strains in a mouse model of disseminated infection. C57BL/6 mice (6 to 8 weeks old) were intravenously injected with 10^7^ bacteria; bacteremia and disseminated spread to the heart, liver, and bladder were monitored at 24 h postinfection. CFU were enumerated and counts were normalized using tissue mass, in grams. Lines and bars show median and interquartile ranges, and data are pooled from 3 independent experiments, each containing *n* = 10 mice, with mutant strains compared using Mann-Whitney U tests to WT colonization data (*, *P* < 0.05; **, *P* < 0.01).

To examine the contribution of Zn efflux to *in vivo* colonization of S. agalactiae, we used a murine model of disseminated infection to monitor tissue and bloodstream burdens (20). Comparisons of the numbers of bacteria recovered from organs of mice that were challenged intravenously with 10^7^ WT or mutant strains are shown in Fig. 4. We detected significantly fewer Δ*czcD* mutant cells in the liver (*P* = 0.046) and bladder (*P* = 0.025) than WT at 24 h postinoculation (Fig. 4A). No differences were observed between counts of the WT and Δ*czcD* mutant in the brain, blood, heart, lungs, kidneys, or spleen (see Fig. S3 in the supplemental material). In addition, significantly fewer Δ*sczA* mutant than WT cells were recovered from the blood (*P* = 0.039) and heart (*P* = 0.032) (Fig. 4B), indicating a modest but statistically significant role for cellular management of Zn via *czcD* and *sczA* in supporting disseminated infection *in vivo*.

10.1128/mSphere.00105-21.3 FIG S3 Mouse model of disseminated infection of WT (grey circles), Δ*czcD* (green diamonds), and Δ*sczA* S. agalactiae (orange triangles). C57BL/6 mice (6 to 8 weeks old) were intravenously injected with 10^7^ bacteria; no significant difference was observed between strains following disseminated spread to the brain, spleen, kidneys, and lungs, monitored at 24 h postinfection. CFU were enumerated, and counts were normalized using tissue mass, in grams. Lines and bars show median and interquartile ranges, and data are pooled from 3 independent experiments each containing *n* = 10 mice. Download FIG S3, TIF file, 0.3 MB. Copyright © 2021 Sullivan et al. 2021 Sullivan et al. https://creativecommons.org/licenses/by/4.0/ This content is distributed under the terms of the Creative Commons Attribution 4.0 International license.

### S. agalactiae Zn stress transcriptome reveals new mediators of resistance.

**FIG 5:**
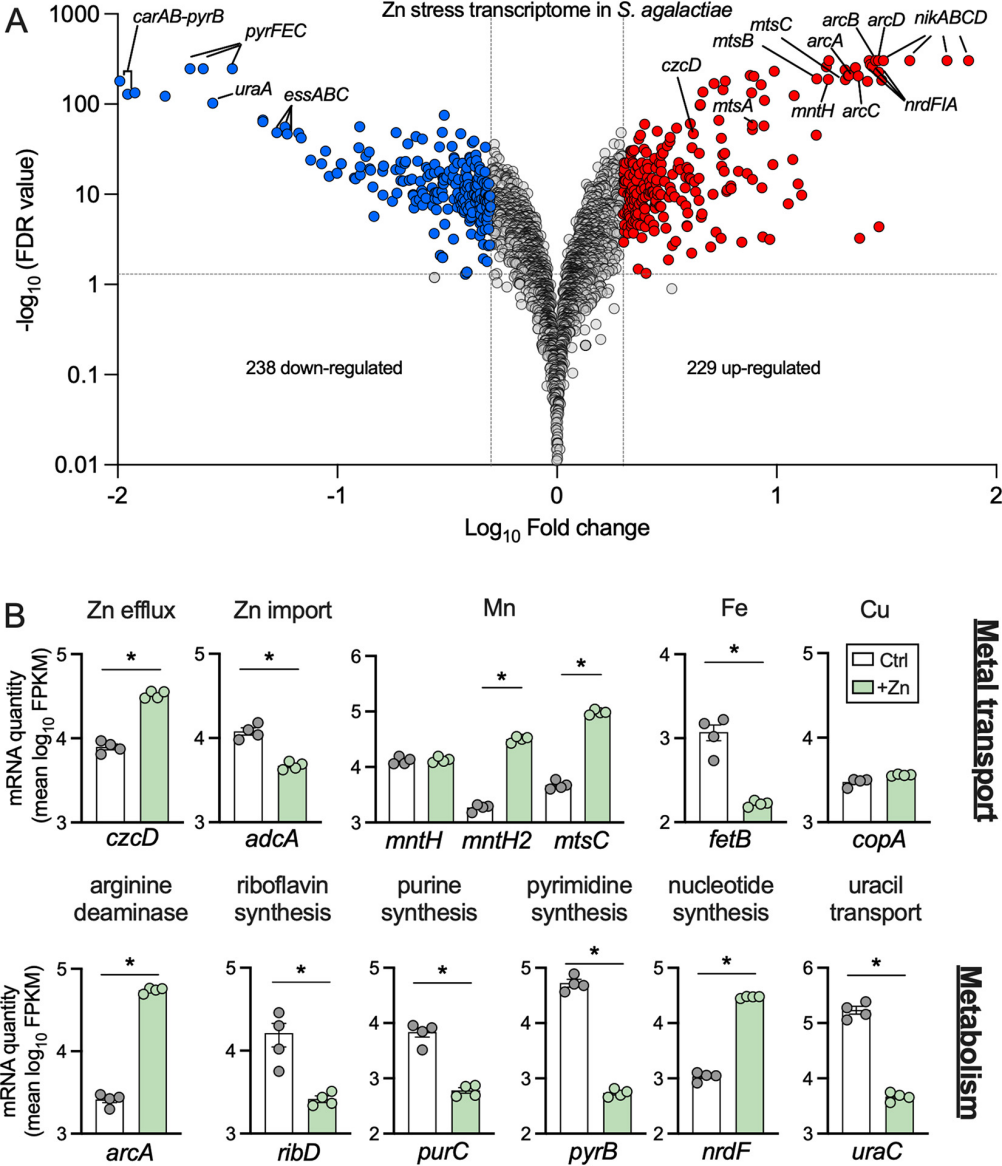
Transcriptomic analysis of S. agalactiae in Zn stress. (A) Volcano plot showing data from RNA-seq analysis of WT S. agalactiae cultures grown in medium containing 0.25 mM supplemental Zn compared to control cultures without supplemental Zn. Transcripts up- or downregulated in response to Zn (*n *=* *4; ±2-fold; FDR <0.05) are highlighted in red and blue, respectively. Dotted lines show false discovery rate (FDR; q-value) and fold change cutoffs. Gray points indicate genes that were unchanged. Selected genes are identified individually with black lines. (B) Expression of selected genes from RNA-seq analyses showing mean fragments per kilobase of transcript per million mapped reads (FPKM) values for each condition, with predicted function as indicated. Data were compared with DESeq2 (*, *P*-adj < 0.05; ±2-fold).

The transcriptome of S. agalactiae in response to Zn stress was used to define the global response of this organism to externally applied Zn. RNA sequencing (RNA-seq) identified 567 genes that were differentially expressed during growth in 0.25 mM Zn (229 up-, 238 downregulated; ±2-fold; adjusted *P* value [*P*-adj], <0.05; *n* = 4 biological replicates) in WT S. agalactiae (Fig. 5A and Table S4). In addition to upregulation of *czcD*, we detected upregulation of several putative nickel and manganese transport loci (*nikABCD*, *mntH2*, and *mtsABC*, respectively) and downregulation of an iron efflux system (*fetAB*) and Zn-importing *adcA* (Fig. 5B). Interestingly, genes encoding transporters for nickel (*nikABCD*), zinc (*adcA*), and Mn (*mntH* and *mtsABC*) were recently implicated in S. agalactiae survival against host-derived calprotectin (21), which mediates Zn starvation, as opposed to Zn intoxication.

10.1128/mSphere.00105-21.8 TABLE S4 The transcriptome of S. agalactiae under Zn stress. Significantly differentially expressed genes (*n = *4 replicates; ±2-fold difference; *Q* < 0.05) identified by RNA-sequencing analysis of WT S. agalactiae grown in THB containing 0.25mM Zn versus THB with no added Zn. Download Table S4, XLSX file, 0.05 MB. Copyright © 2021 Sullivan et al. 2021 Sullivan et al. https://creativecommons.org/licenses/by/4.0/ This content is distributed under the terms of the Creative Commons Attribution 4.0 International license.

**FIG 6:**
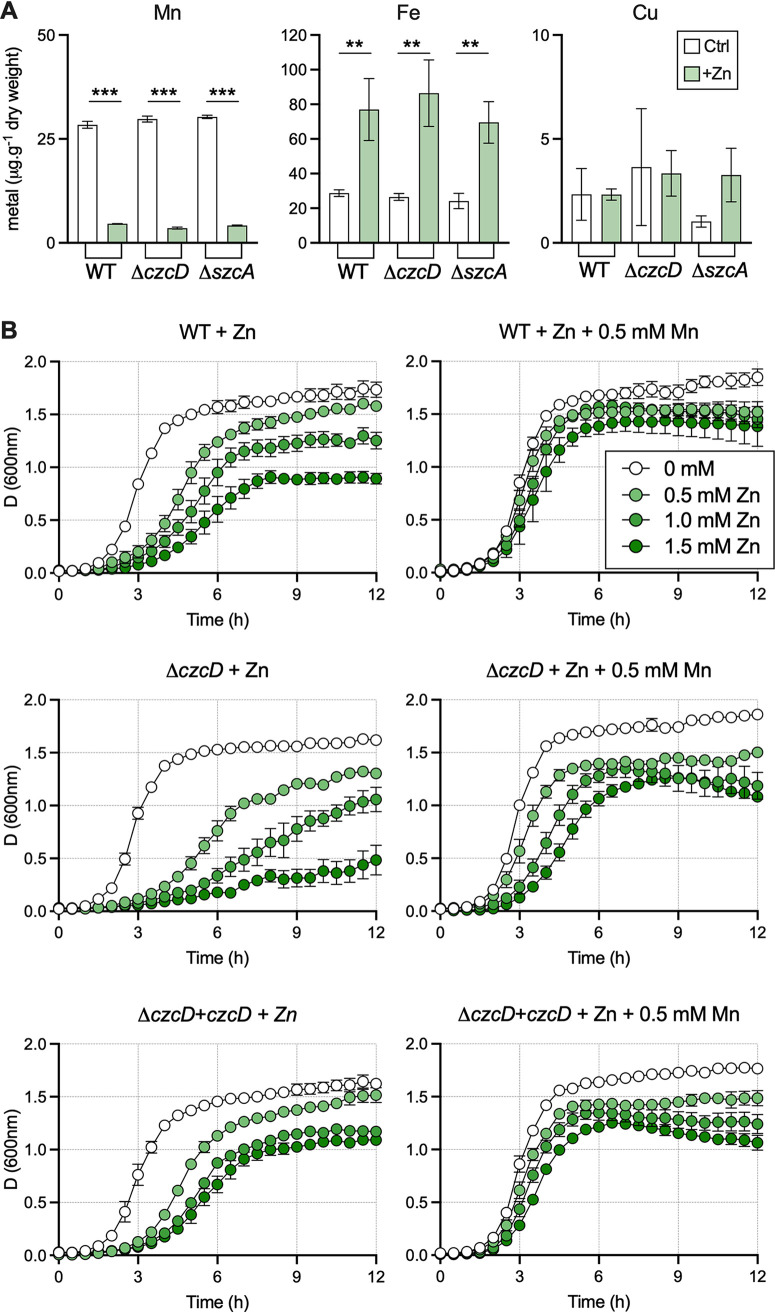
Intracellular metal accumulation during Zn stress and Mn rescue. (A) Intracellular accumulation of Mn, Fe, and Cu was compared with and without Zn supplementation in WT, Δ*czcD*, and Δ*sczA* S. agalactiae strains using ICP-OES. Bars show means and SEM from 3 to 4 independent experiments and were compared by ordinary one-way ANOVA with Holm-Sidak multiple comparisons (**, *P* < 0.01; ***, *P* < 0.001). (B) Manganese supplementation rescues Zn toxicity in S. agalactiae. Growth comparisons of WT, Δ*czcD*, and Δ*czcD+czcD* strains in THB or THB supplemented with 0.5, 1.0, or 1.5 mM Zn compared to the same medium but additionally supplemented with 0.5 mM Mn. Data are means and SEM from three independent experiments.

To provide functional insight into the observed changes in metal transporters, we analyzed cellular metal content of S. agalactiae during Zn stress for other second-row transition metals, including Mn, Fe, Ni, and Cu. We saw significant reductions in Mn levels, enhanced cellular Fe, and no difference in Cu levels, and Ni was below the detection limit (0.8 ppm) (Fig. 6A) for cultures of WT bacteria under Zn stress (0.25 mM) compared to nonsupplemented controls (THB medium only); we observed no difference in the levels of Mn, Fe, and Cu in comparing the WT to the Δ*czcD* or Δ*sczA* mutant (Fig. 6A). Given the reduction in Mn during conditions of Zn stress, we undertook experiments in which we supplied Mn in excess (0.5 mM) in addition to supplemental Zn and observed a complete rescue of the Zn sensitivity phenotypes of both the WT and Δ*czcD* strains (Fig. 6B).

In addition to metal transporters, we detected modulation of several metabolism-related gene clusters that have not previously been linked to Zn stress in bacteria, encompassing *de novo* nucleotide synthesis and import systems (*carAB*, *pyrB*, *pyrFEC*, *pur* genes, *uraA*, *nrdFIA*, and *guaC*) and riboflavin synthesis (*ribDEAH*) loci (Fig. 5 and Table S4). Interestingly, a putative arginine deaminase system (ADI), encoded by the *arcABCD* genes, was significantly upregulated (21- to 29-fold) under Zn intoxication (*arcA* highlighted in Fig. 5B). The *arcABC* genes encode arginine deaminase (ArcA), ornithine carbamoyltransferase (ArcB), and carbamate kinase (ArcC), with *arcD* encoding an ornithine/arginine antiporter. This system functions to produce ammonia and ATP from the conversion of arginine to ornithine, as characterized in S. pneumoniae (22), and is responsive to numerous stimuli (23).

**FIG 7:**
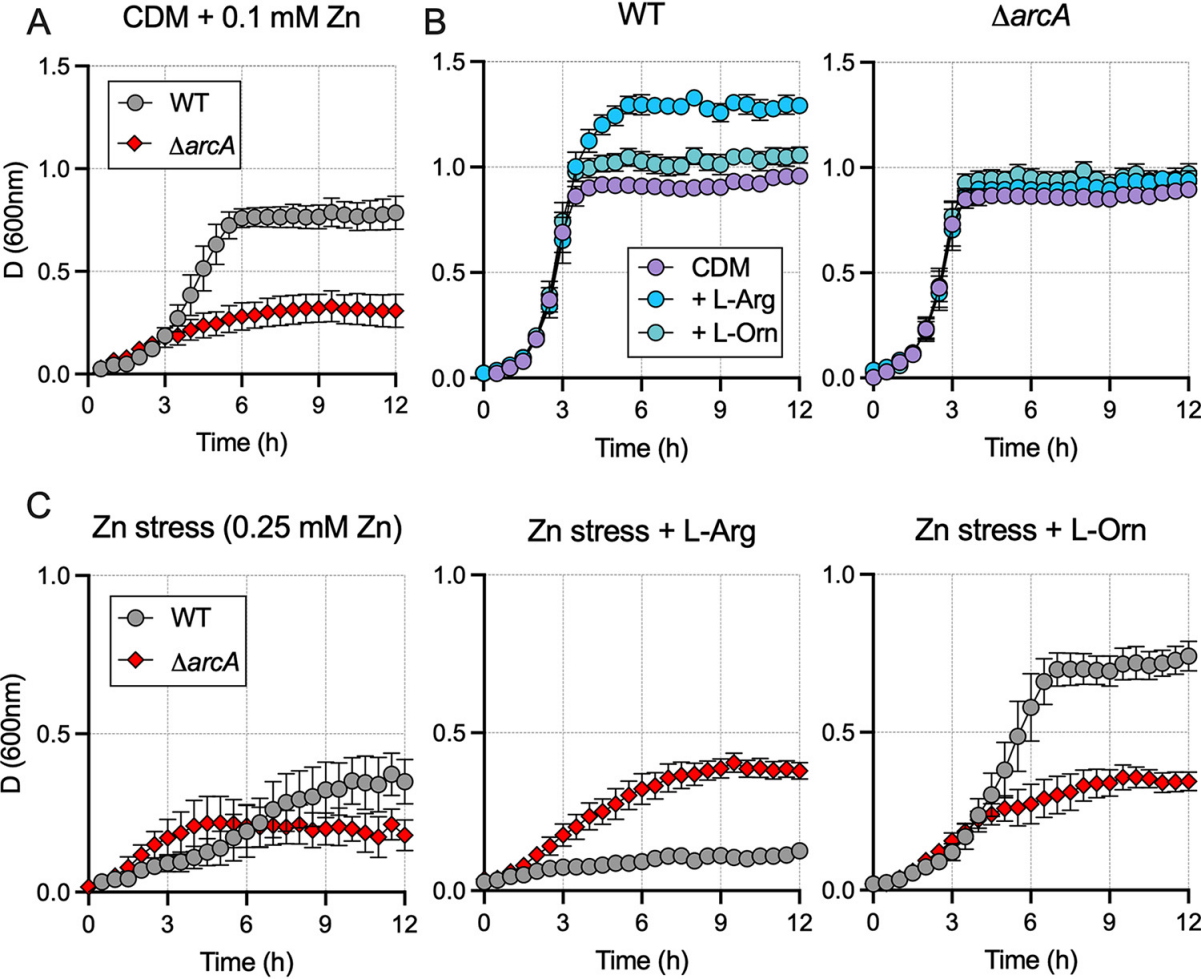
Comparisons of the growth of WT S. agalactiae and an Δ*arcA* isogenic mutant. (A) Cultures of WT and Δ*arcA* S. agalactiae were grown in nutrient-limited CDM under moderate Zn stress (±0.1 mM Zn). (B) Cells were grown in CDM (without Zn) or CDM supplemented with 10 mM l-arginine or 10 mM l-ornithine. l-Arginine, but not l-ornithine, supplementation increases growth yield and final culture densities in the WT but not the Δ*arcA* strain. (C) WT and Δ*arcA* S. agalactiae were grown under conditions of severe Zn stress (+0.25 mM Zn) in CDM, with or without supplementation with 10 mM l-arginine or 10 mM l-ornithine. l-Ornithine rescues Zn sensitivity compared to 10 mM l-arginine or nonsupplemented controls. Data shown are mean measurements of attenuance (*D*; at 600 nm) from 3 to 4 independent experiments.

We generated an isogenic Δ*arcA* mutant to examine a potential role for ADI in Zn stress resistance in S. agalactiae. Comparing the growth of the Δ*arcA* mutant to the WT under nutrient-limiting CDM plus 0.1 mM Zn (conditions in which Δ*czcD* and Δ*sczA* strains were perturbed) revealed significant attenuation of the Δ*arcA* strain for growth under Zn stress (Fig. 7A and Fig. S4). We used CDM, which normally contains 0.39 mM l-arginine, to confirm that S. agalactiae *arcA* has a role in arginine metabolism. We monitored growth of the WT compared to the Δ*arcA* mutant using CDM with or without supplementation with 10 mM l-arginine or l-ornithine (Fig. 7B). Final biomass yield was significantly elevated in WT cultures supplemented with l-arginine compared to the Δ*arcA* mutant, which showed no enhancement of growth with added l-arginine. Next, we reasoned that the products of arginine deamination confer an advantage to surviving Zn intoxication in S. agalactiae. To examine this, we analyzed growth in CDM supplemented with 0.25 mM Zn (severely inhibitory to WT) and supplied either l-arginine or l-ornithine, as described above. Strikingly, we saw that addition of l-ornithine rescued the Zn sensitivity phenotype of WT S. agalactiae during intoxication by 0.25 mM Zn, a phenomenon that was not observed in the Δ*arcA* strain (Fig. 7C). Addition of l-arginine was modestly inhibitory to the WT during intoxication by 0.25 mM Zn (Fig. 7C).

10.1128/mSphere.00105-21.4 FIG S4 Comparison of the growth of WT S. agalactiae and an Δ*arcA* isogenic mutant under Zn stress in nutritive THB medium with or without 1 mM Zn versus control cultures without supplemental Zn. In THB without Zn, a modest but statistically significant difference between WT and Δ*arcA* strains was observed. Supplementation with 1 mM Zn impacted growth rate and final biomass yield of the Δ*arcA* mutant in a fashion similar to that observed in CDM (Fig. 6). Data shown are mean measurements of attenuance (*D*; at 600nm) from 3 to 4 independent experiments. Bars show SEM, and results were compared using area under the concentration-time curve followed by Student’s *t* test (*, *P* < 0.05; **, *P* < 0.01). Download FIG S4, TIF file, 0.2 MB. Copyright © 2021 Sullivan et al. 2021 Sullivan et al. https://creativecommons.org/licenses/by/4.0/ This content is distributed under the terms of the Creative Commons Attribution 4.0 International license.

Finally, S. agalactiae modulated several genes encoding classical virulence and/or immunogenic factors in response to Zn intoxication, including the *cyl* gene cluster *cylXDG-acp-cylZABEFIJK* (encoding β-hemolysin/cytolysin) (2.5- to 4.3-fold down), *lrrG* (25-fold up, leucine-rich repeat protein), and *essABC* (11- to 22-fold down, type VII secretion system) (Table S4). We also note that ∼20% of all transcripts (114/567) that were differentially regulated in response to Zn intoxication are predicted to encode hypothetical proteins of unknown function, some of which were up- or downregulated up to ∼29-fold. These observations represent a significant pool of targets for further studies in dissecting bacterial responses to Zn stress.

## DISCUSSION

**FIG 8:**
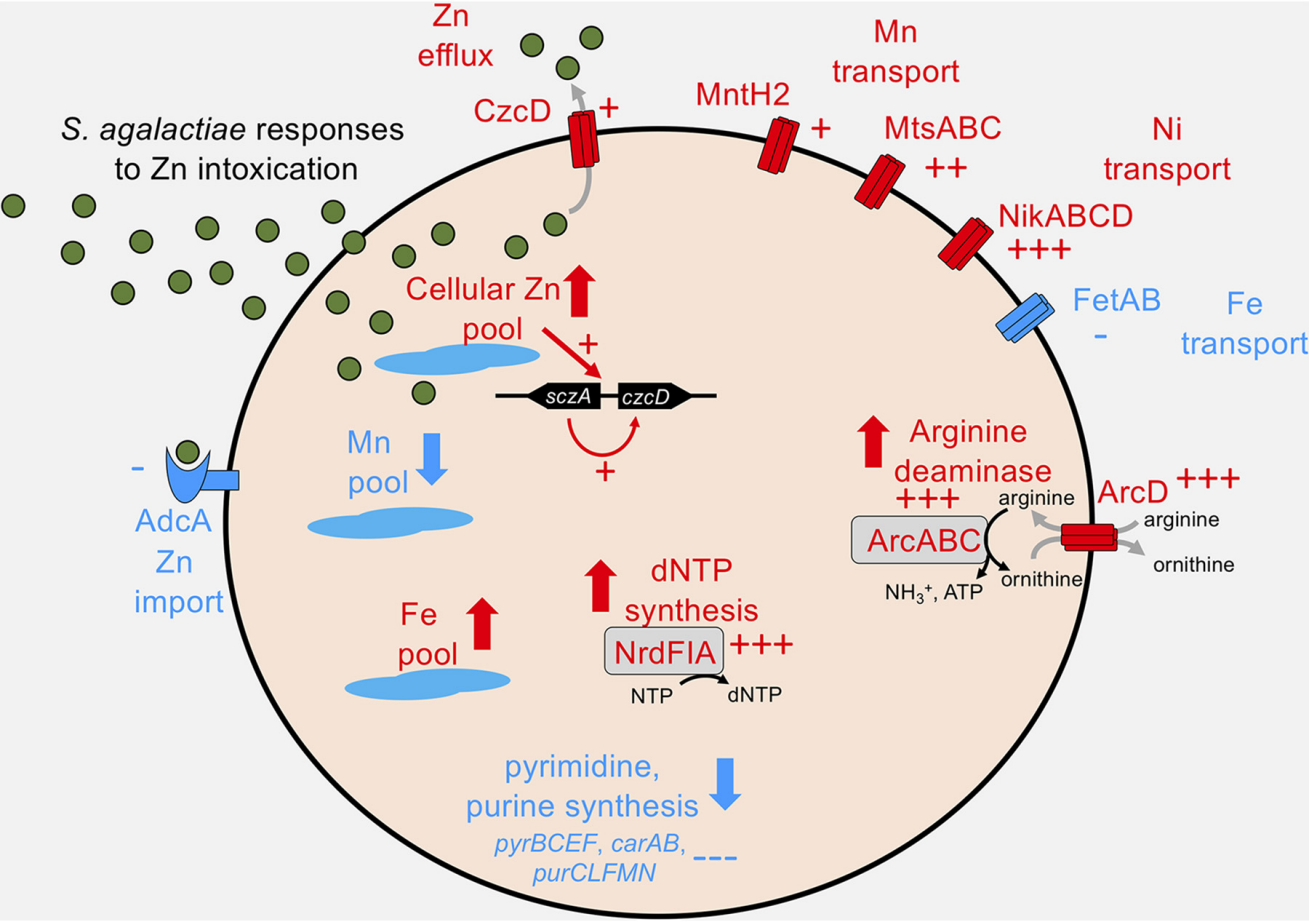
Summary of selected S. agalactiae responses to Zn intoxication. S. agalactiae senses elevated intracellular Zn to coordinate enhanced CzcD expression via the Zn-sensing SczA regulator. Zn stress results in a number of notable changes, including differential expression of metal transporters, altered Zn, Mn, and Fe cellular pools, and metabolic pathways encompassing arginine deaminase, *de novo* purine and pyrimidine syntheses, and dNTP synthesis. Red coloring and arrows indicate upregulation (+, 2- to 10-fold; ++, 10- to 20-fold, +++, >20-fold) or downregulation (−, 2 to 10-fold; −, >20-fold) of a transcript or process.

This study establishes a key role for cellular management of intracellular Zn levels in S. agalactiae via *czcD*, *sczA*, and additional mediators, including *arcA*, in conferring an ability to resist Zn intoxication to the bacteria. These findings provide new insight into the molecular mechanisms of virulence used by this pathogen to survive under stressful environmental conditions resulting from elevated metal ion levels, such as those within phagocytes during infection in a host. In characterizing the Zn efflux systems of S. agalactiae in detail, the findings of the present study support prior findings from work on other streptococci (17, 18); for example, our characterization of regulatory functions of *sczA* that enable cellular management of Zn in S. agalactiae are consistent with prior findings reported for S. pyogenes (17). These prior observations include that Zn stress upregulates Zn efflux via *czcD* and shuts down Zn uptake via *adcA* (24), with direct effects on the control of intracellular Zn content. Importantly, the Zn stress-response global transcriptome of S. agalactiae defined in the current study also elucidates other metal ion transporters and novel additional targets that have not previously been linked to Zn intoxication in any bacteria. A model of the Zn stress response in S. agalactiae based on the findings of this study is shown in Fig. 8.

Systems for Zn acquisition in S. agalactiae enable survival of the organism under Zn-deficient conditions (9) and confer resistance to calprotectin-mediated metal ion starvation (21), which acts to withhold multiple metals, such as Mn and Zn, from bacteria in the host (25). Our Zn intoxication transcriptional analyses identified numerous metal transporters that are differentially regulated, including *nikABCD* and *mtsABC*, predicted to transport Ni and Mn, respectively. Experiments in which we used supplemental Mn in the growth media revealed a rescue of the Zn sensitivity of the *czcD* mutant, further pointing to potentially important roles for such transporters. Given that Ni and Mn import genes have been implicated in bacterial survival during calprotectin stress (21), it is possible that *nik* and *mts* genes respond to altered cellular levels of other metals (rather than Zn) that occur as a consequence of Zn intoxication.

In our study, levels of cellular Mn were perturbed during Zn intoxication. This coincided with corresponding upregulation of *mntH2* and *mtsABC* transcription, encoding putative Mn transporters. Interestingly, some, but not all, S. agalactiae strains possess two genes encoding proteins homologous to NRAMP-type MntH transporters. *mntH* (CHF17_00875, ASZ01148.1) contributes to acid stress responses (26), but a role for *mntH2* (CHF17_02002, ASZ02237.1) has not been determined. In addition, S. agalactiae elevated Fe levels in response to Zn stress, coinciding with downregulation of a putative Fe export system encoded by *fetA-fetB*. In contrast, Cu levels and expression of *copA* (encoding a putative Cu transporter) were unaffected in S. agalactiae grown under Zn stress conditions. Collectively, these data show broader dysregulation of metal management in S. agalactiae undergoing Zn intoxication beyond Zn itself, encompassing Mn and Fe, consistent with a prior report on pneumococci (14). Future examination of the roles of the Ni, Mn, Fe, and Cu transport in S. agalactiae in the setting of bacterial metal stress and in models of infection would be of interest.

The finding that numerous core metabolic pathways in S. agalactiae are impacted by Zn stress, including some related to synthesis of purines, pyrimidines, riboflavin, and deoxynucleoside triphosphates, underscores the critical nature of Zn management for maintenance of basic cellular processes in the bacteria. The arginine deaminase system was among the most differentially regulated gene clusters, undergoing 20- to 30-fold enhanced expression during Zn stress. Remarkably, *arcA*-deficient S. agalactiae exhibited heightened sensitivity to Zn stress, establishing a novel role for this system in streptococcal resistance to metal ion intoxication. The precise molecular mechanisms that underpin how this system renders the bacterial cell resistant to Zn intoxication requires further definition, but arginine deaminase typically converts arginine to ammonia, ATP, and ornithine; overproduction of one of these may support bacterial survival during Zn stress. Consistent with this theory, we observed rescue of Zn sensitivity in S. agalactiae by supplementation with ornithine but not arginine. This interesting observation will require further examination in future work. A recent study identified arginine deaminase as a key factor in resistance to antibiotics and biofilm formation in S. pyogenes (27). Numerous prior studies have reported elevated expression of *arc* genes in S. agalactiae in response to acid stress (28), human serum (29), blood (30), or amniotic fluid (31). This suggests important but yet-to-be defined roles for arginine deaminase in the virulence of S. agalactiae. Together with the findings of the current study, these observations suggest that arginine deaminase supports the ability of S. agalactiae to respond to diverse stressors in addition to Zn intoxication.

Analysis of the role for Zn efflux systems of S. agalactiae in pathogenesis showed no contribution to bacterial survival in macrophages. This finding is surprising in the context of a prior study of S. pneumoniae *sczA* (that is functionally analogous to S. agalactiae *sczA*), which reported a major role for Zn cellular management via *sczA* in the intracellular survival of the bacteria in human macrophages (19). We confirmed the generation of a robust Zn mobilization response in the host cells following S. agalactiae infection but observed equivalent fitness of mutants for Zn management compared to the WT for survival in murine and human macrophages. Considering the conserved nature of *szcA* between streptococci, it is plausible that these mutants are attenuated in other phagocytes, such as neutrophils, as described for S. pyogenes (17), noting a need for further examination of differences in metal ion resistance phenotypes among streptococcal species.

The discovery of attenuation for colonization in a mouse model of disseminated infection in the S. agalactiae mutants in Zn management systems in this study suggests a role for Zn stress in disease pathogenesis due to this organism. The attenuations observed for the *czcD* and *sczA* mutants in the liver and bladder and the blood and heart, respectively, were not dramatic according to the tissue bacterial loads subsequent to blood challenge; however, the attenuations were statistically significant. Prior studies have shown more dramatic effects of *czcD* and *sczA* mutations in reducing bacterial virulence in mouse models of infection. For example, *czcD* mutation rendered S. pyogenes nonlethal in a subcutaneous infection model in mouse (17). *czcD* mutation attenuated S. pneumoniae 2.6-fold for survival in the lungs of mice following intranasal challenge compared to WT bacteria (32). Evaluating the current findings in the context of these prior studies highlights the differences in experimental designs, *viz.*, types of infection models, bacterial species, and outcome measures that would likely influence any potential degree of attenuation in mutants (17, 19, 32). For example, dietary zinc can influence bacterial colonization and/or survival, as shown in a murine model of infection with pneumococcus (32); in our study, we used a standard fixed-formulation diet (trace mineral content, in mg/kg of body weight: Mg, 100; Fe, 70; Cu, 16; I, 0.5; Mg, 70; Zn, 60; Mo, 0.5; Se, 0.1), and it possible that modifications to the trace mineral content of this diet will lead to different levels of recovery of bacteria in our model. We recognize this as a limitation of our study. More broadly, this highlights the importance of evaluating the roles of individual mediators, such as CzcD, SczA, and ArcA, in appropriate experimental models that are designed to parallel natural infection in the human host. For S. agalactiae, for example, exploration of the role of resistance to Zn stress in colonization of the female genital tract (33) and the brain (34) would be of interest given the propensity of this pathogen to cause these infections in humans (35).

In conclusion, this study identifies new mediators of Zn cellular management in S. agalactiae and shows that resistance to Zn stress in this pathogen contributes to colonization in the host. Future examination of these mediators, and their role in the survival of S. agalactiae and other bacterial pathogens in relevant settings of infection, will be important to more fully understand microbial resistance to metal intoxication and its influence on virulence and pathogenesis.

## MATERIALS AND METHODS

### Bacterial strains, plasmids, and growth conditions.

S. agalactiae, Escherichia coli, and plasmids used are listed in Table S1 in the supplemental material. S. agalactiae was routinely grown in Todd-Hewitt broth (THB) or on TH agar (1.5%, wt/vol). E. coli was grown in lysogeny broth (LB) or on LB agar. Media were supplemented with antibiotics (spectinomycin [Sp], 100 μg/ml; chloramphenicol [Cm], 10 μg/ml), as indicated. Growth assays used 200-μl culture volumes in 96-well plates (Greneir) sealed using Breathe-Easy membranes (Sigma-Aldrich) and measured attenuance (*D*; at 600 nm) using a ClarioSTAR multimode plate reader (BMG Labtech). Attenuance measurements used well-scan mode with a 3-mm, 5-by-5 scan matrix, 5 flashes per point, path length correction of 5.88 mm, and 300-rpm agitation every 30 min. Media for growth assays were THB and a CDM (9), modified in the current study to include final concentrations of 1 g/liter glucose, 0.11 g/liter pyruvate, and 50 μg/liter l-cysteine. For metal ion assays, the media were supplemented with Zn (supplied as ZnSO_4_), Mn (supplied as MnCl_2_), l-arginine, or l-ornithine as indicated. For attenuance baseline correction, control wells without bacteria were included for Zn in medium alone.

10.1128/mSphere.00105-21.5 TABLE S1 Bacterial strains and plasmids used in this study. Download Table S1, DOCX file, 0.02 MB. Copyright © 2021 Sullivan et al. 2021 Sullivan et al. https://creativecommons.org/licenses/by/4.0/ This content is distributed under the terms of the Creative Commons Attribution 4.0 International license.

### DNA extraction and genetic modification of S. agalactiae.

Plasmid DNA was isolated using miniprep kits (Qiagen), with modifications for S. agalactiae as described elsewhere (36). Electroporation of S. agalactiae and selection of transformants was performed as described previously (37). Deletion of *czcD* (CHF17_00567/CHF17_RS02855, ASZ00854.1) was performed by allelic exchange with a Cm cassette using pHY304aad9 as described previously (37). Briefly, ∼400- to 500-bp regions upstream and downstream of *czcD* were amplified using primers (Table S2) carrying 21- to 23-bp overlapping sequence complementary to the Cm cassette of pLZ12 to facilitate fusion of these amplicons to chloramphenicol acetyltransferase by 3-way PCR. The subsequent product was cloned into pHY304aad9. Constructs for *sczA* and *arcA* deletions were made with DNA that was first synthesized in pUC57 by GenScript (USA), encompassing 500 bp of sequence upstream of either *sczA* or *arcA* fused to 500 bp of downstream sequence (Fig. S1) prior to subcloning into pHY304aad9. In-frame, markerless deletions in *sczA* and *arcA* were generated similarly by exploiting temperature selection using pHY304aad9, followed by loss of Sp resistance to identify double-crossover mutants. Constructs, complement plasmids, and primers are listed in Table S2. Mutants were validated by PCR using primers external to the mutation site and DNA sequencing.

10.1128/mSphere.00105-21.1 FIG S1 To make markerless mutations in *sczA* (A) and *arcA* (B) of S. agalactiae 874391 (44), the sequence was synthesized by GenScript (USA) in pUC57 and flanked by XhoI and BamHI restriction sites (black). In both cases, 500 bp of sequence upstream (blue) was combined with 500 bp of downstream sequence (orange), separated by an SalI site (black; for optional cassette insertion), such that deletion by allelic exchange resulted in replacement of codon 5-172 of SczA or 13-404 of ArcA, with a 6-bp SalI site (encoding Val-Asp), removing 96% and 95% of the coding sequences of *sczA* and *arcA*, respectively, and creating in-frame, markerless deletions in the S. agalactiae 874391 genome. Download FIG S1, TIF file, 0.9 MB. Copyright © 2021 Sullivan et al. 2021 Sullivan et al. https://creativecommons.org/licenses/by/4.0/ This content is distributed under the terms of the Creative Commons Attribution 4.0 International license.

10.1128/mSphere.00105-21.6 TABLE S2 Oligonucleotides used in this study. Download Table S2, DOCX file, 0.01 MB. Copyright © 2021 Sullivan et al. 2021 Sullivan et al. https://creativecommons.org/licenses/by/4.0/ This content is distributed under the terms of the Creative Commons Attribution 4.0 International license.

### Expression system for mCherry in S. agalactiae.

Plasmid pGU2665 was designed for expressing mCherry in *trans* in S. agalactiae from the pCP25 promoter (38) cloned into pDL278 (39) (Fig. S2). Plasmid DNA was manipulated, and ligation reactions were performed essentially as described elsewhere (36). We verified pGU2665 by restriction analysis and sequencing using primers listed in Table S2. Sequence reads were mapped and assembled using Sequencher software. Plasmid stability assays were performed as described previously (36).

10.1128/mSphere.00105-21.2 FIG S2 Construction of mCherry-S. agalactiae for use in *in vitro* macrophage infection assays. (a) Map of plasmid pGU2665 for expressing mCherry in S. agalactiae, illustrating an ∼1.9-kb EcoRI insert (indicated by E notations) containing mCherry driven by the constitutive CP25 promoter (36). (b) Visualization of live S. agalactiae cells containing pGU2665; images were captured by detecting mCherry fluorescence emission (×100 magnification). Attenuance (c) and relative mCherry fluorescence units (d), monitored during growth of S. agalactiae strains with (mCherry-S. agalactiae) or without (WT-S. agalactiae) plasmid pGU2665 (*n = *2). Download FIG S2, TIF file, 0.7 MB. Copyright © 2021 Sullivan et al. 2021 Sullivan et al. https://creativecommons.org/licenses/by/4.0/ This content is distributed under the terms of the Creative Commons Attribution 4.0 International license.

### RNA extraction, qRT-PCR.

For Zn exposure experiments, 1-ml overnight THB cultures were back-diluted 1/100 in 100 ml of THB (prewarmed at 37°C in 250-ml Erlenmeyer flasks) supplemented with 0.25, 0.5, 1.0, or 1.5 mM Zn. Cultures were grown with shaking (200 rpm) at 37°C; after exactly 2.5 h, 10- to 50-ml volumes containing approximately 500 million mid-log-phase bacteria were harvested; RNA was preserved and isolated as described previously (40). RNA quality was analyzed by RNA LabChip using GX Touch (Perkin Elmer). RNA (1,000 ng) was reverse transcribed using Superscript IV according to the manufacturer’s instructions (Life Technologies), and cDNA was diluted 1:50 in water prior to quantitative PCR. Primers (Table S2) were designed using Primer3 Plus (41, 42) to quantify transcripts using Universal SYBR green supermix (Bio-Rad) and a Quantstudio 6 Flex (Applied Biosystems) system in accordance with MIQE guidelines (43). Standard curves were generated using five point serial dilutions of genomic DNA (5-fold) from WT S. agalactiae 874391 (44). Expression ratios were calculated using threshold cycle (*C_T_*) values and primer efficiencies, as described elsewhere (45), using *dnaN*, encoding DNA polymerase III β-subunit as a housekeeper gene.

### Whole bacterial cell metal content determination.

Metal content in cells was determined as described previously (14), with minor modifications. Cultures were prepared essentially as described for RNA extraction and qRT-PCR, with the following modifications. THB medium was supplemented with 0.25 mM ZnSO_4_ or not supplemented (Ctrl), and, following exposure for 2.5 h, bacteria were harvested by centrifugation at 4,122 × *g* at 4°C. Cell pellets were washed 3 times in phosphate-buffered saline (PBS) plus 5 mM EDTA to remove extracellular metals, followed by 3 washes in PBS. Pelleted cells were dried overnight at 80°C and resuspended in 1 ml of 32.5% nitric acid and incubated at 95°C for 1 h. The metal ion-containing supernatant was collected by centrifugation (14,000 × *g*, 30 min) and diluted to a final concentration of 3.25% nitric acid for metal content determination using inductively coupled plasma optical emission spectroscopy (ICP-OES). ICP-OES was carried out on an Agilent 720 ICP-OES with an axial torch, OneNeb concentric nebulizer, and Agilent single-pass glass cyclone spray chamber. The power was 1.4 kW with 0.75 liters/min nebulizer gas, 15 liters/min plasma gas, and 1.5 liters/min auxiliary gas flow. Zn was analyzed at 213.85 nm, Cu at 324.75 nm, Fe at 259.94 nm, and Mn at 257.61 nm, with detection limits at <1.1 ppm. The final quantity of each metal was normalized using dry weight biomass of the cell pellet prior to nitric acid digestion, expressed as micrograms per gram of dry weight. Baseline concentrations of Zn, Fe, Mn, and Cu in standard THB or CDM used in this study were determined from 3 independent assays (Table S3).

10.1128/mSphere.00105-21.7 TABLE S3 Metal ion concentrations in basal growth media. Download Table S3, DOCX file, 0.01 MB. Copyright © 2021 Sullivan et al. 2021 Sullivan et al. https://creativecommons.org/licenses/by/4.0/ This content is distributed under the terms of the Creative Commons Attribution 4.0 International license.

### RNA sequencing and bioinformatics.

Cultures were prepared as described above for RNA extraction and qRT-PCR. RNase-free DNase-treated RNA that passed Bioanalyzer 2100 (Agilent) analysis was used for RNA sequencing (RNA-seq) using the Illumina NextSeq 500 platform. We used TruSeq library generation kits (Illumina, San Diego, CA). Library construction consisted of random fragmentation of the poly(A) mRNA, followed by cDNA synthesis using random primers. The ends of the cDNA were repaired and A-tailed, and adaptors were ligated for indexing (with up to 12 different barcodes per lane) during the sequencing runs. The cDNA libraries were quantitated using quantitative PCR in a Roche LightCycler 480 with the Kapa Biosystems kit (Kapa Biosystems, Woburn, MA) prior to cluster generation. Clusters were generated to yield approximately 725K to 825K clusters/mm^2^. Cluster density and quality were determined during the run after the first base addition parameters were assessed. We ran single-end 75-bp sequencing runs to align the cDNA sequences to the reference genome. For data preprocessing and bioinformatics, STAR (version 2.7.3a) was used (parameters used were –outReadsUnmapped Fastx –outSAMtype BAM SortedByCoordinate –outSAMattributes All) to align the raw RNA sequencing fastq reads to the WT S. agalactiae 874391 reference genome (44). HTSeq-count, version 0.11.1 (parameters used were -r pos -t exon -i gene_id -a 10 -s no -f bam), was used to estimate transcript abundances (46). DESeq2 was then used to normalize and test for differential expression and regulation following their vignette. Genes that met certain criteria (i.e., fold change of more than ±2.0, *q* value of <0.05) were accepted as significantly altered (47).

### Mammalian cell culture.

J774A.1 murine macrophages or U937 human monocyte-derived macrophages (MDMs) were grown in RPMI and seeded (10^5^) into the wells of a 96-well tissue culture-treated plate (Falcon) essentially as described elsewhere (48, 49), except that U937 MDMs were differentiated by exposure to 50 ng/ml phorbol 12-myristate 13-acetate (PMA) for 48 h and cells subsequently rested in media without PMA for 72 h to enhance morphological and phenotypic markers of MDMs (50). A multiplicity of infection (MOI) of 100 bacteria to macrophage for 1 h was used in RPMI without antibiotics. Nonadherent bacteria were removed by five washes of 200 μl PBS using a Well Wash Versa (Thermo Scientific). RPMI containing 250 U/ml penicillin, streptomycin (Gibco), and 50 μg/ml gentamicin (Sigma-Aldrich) was used for antibiotic protection assays to quantify intracellular bacteria as described previously (49). At 1 h, 24 h, or 48 h after infection, monolayers were washed five times with 200 μl PBS and lysed by brief exposure to 50 μl of 2% trypsin and 0.02% Triton X-100 (10 min) prior to dilution with 150 μl PBS and estimation of the number of CFU per milliliter by serial dilution and plate counts on agar.

### Fluorescence microscopy.

Fifty thousand J774A.1 cells were seeded into 8-well LabTek II chamber slides (Nunc) and infected with mCherry-S. agalactiae for 1 h, followed by washing and application of antibiotics according to the antibiotic protection assay as described above. Then, 24 h later, the infected cells were subjected to three washes of 200 μl PBS and fixed for 15 min at 37°C using 4% (wt/vol) paraformaldehyde. Monolayers were stained for Zn^2+^ using 5 μM FluoZin-3 AM (Life Technologies) for 30 min at 37°C and subsequently for DNA using Hoechst 33258 for 5 min. Fixed, stained cells were washed twice in PBS and mounted using *n*-propyl gallate (n-pg) mounting medium (0.2% n-pg in 9:1 glycerol-PBS). mCherry-S. agalactiae was visualized using a Zeiss AxioImager.M2 microscope (Carl Zeiss MicroImaging) fitted with Plan-Apochromat X20/0.8 and X63/1.40 lens objectives and an AxioCam MRm Rev.3 camera. Images of cells were captured with 63HE, 44, and 49 filter sets (to detect mCherry [587 nm, 610 nm], FluoZin-3 [494 nm, 518 nm], and Hoechst 33258 [352 nm, 461 nm] fluorescence, respectively, with excitation and emission spectra listed consecutively for each) and Zen Pro (version 2) software.

### Animals and ethics statement.

Virulence was tested using a mouse model of disseminated infection based on intravenous challenge as described elsewhere (20). Briefly, an inoculum of 10^7^ S. agalactiae in 200 μl of PBS (pH 7.4) was delivered to each mouse via the lateral tail vein using a 1-ml syringe connected to a 27-guage by 1.25-inch regular wall needle. This study was carried out in accordance with the guidelines of the Australian National Health and Medical Research Council. The Griffith University Animal Ethics Committee reviewed and approved all experimental protocols for animal usage according to the guidelines of the National Health and Medical Research Council (approval MSC/01/18/AEC).

### Statistical methods.

All statistical analyses used GraphPad Prism V8 and are defined in the respective figure legends. Statistical significance was accepted at *P* values of ≤0.05.

### Data availability.

Raw and processed data were deposited in the Gene Expression Omnibus (accession no. GSE161127 for wild-type [WT] S. agalactiae 874391 during Zn intoxication; GSE167894 for WT S. agalactiae 874391 control).
