# Supplementary figures and images for: Cellular Management of Zinc in Group B Streptococcus Supports Bacterial Resistance against Metal Intoxication and Promotes Disseminated Infection
Source: mSphere. 2021 May 19;6(3):e00105-21. doi: 10.1128/mSphere.00105-21 (PMC8265624; doi:10.1128/mSphere.00105-21)

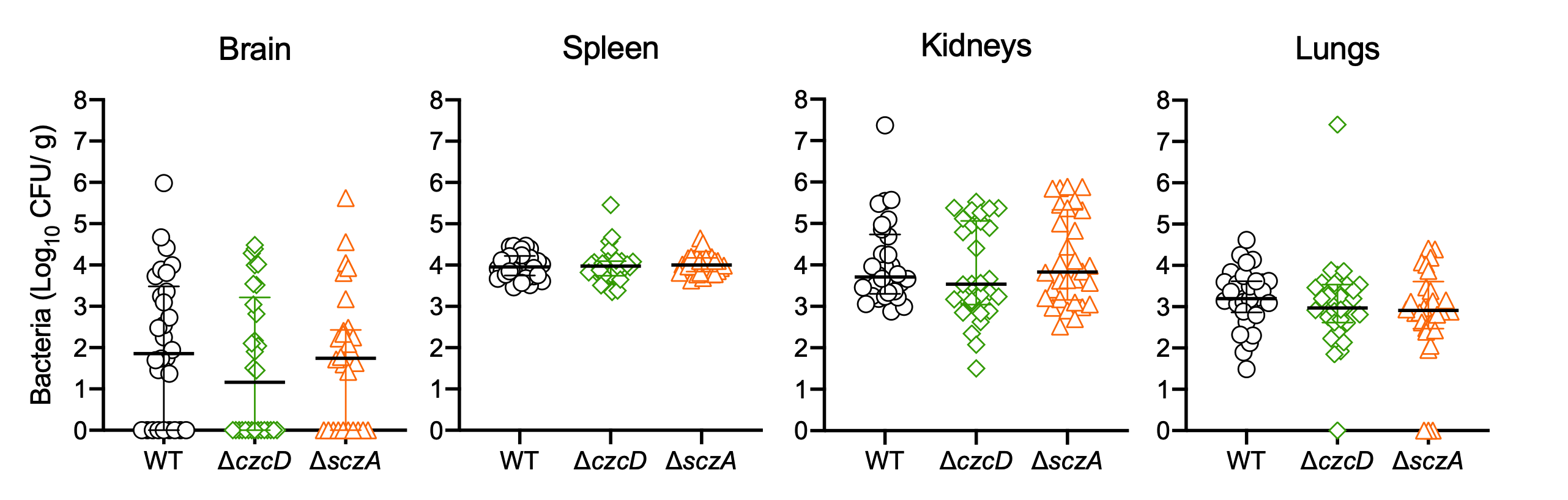

Supplement: FIG S3 [file msphere.00105-21-sf003.tif]

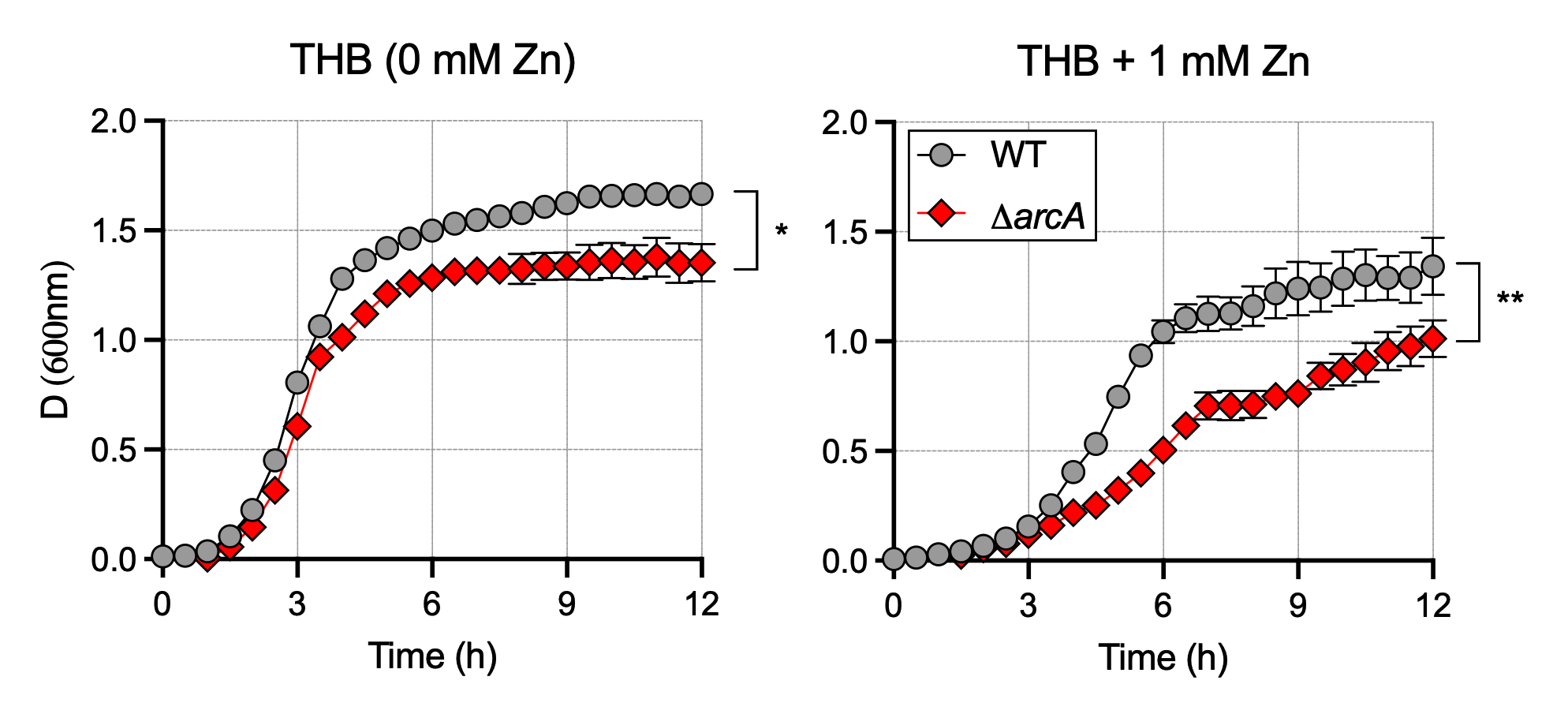

Supplement: FIG S4 [file msphere.00105-21-sf004.tif]

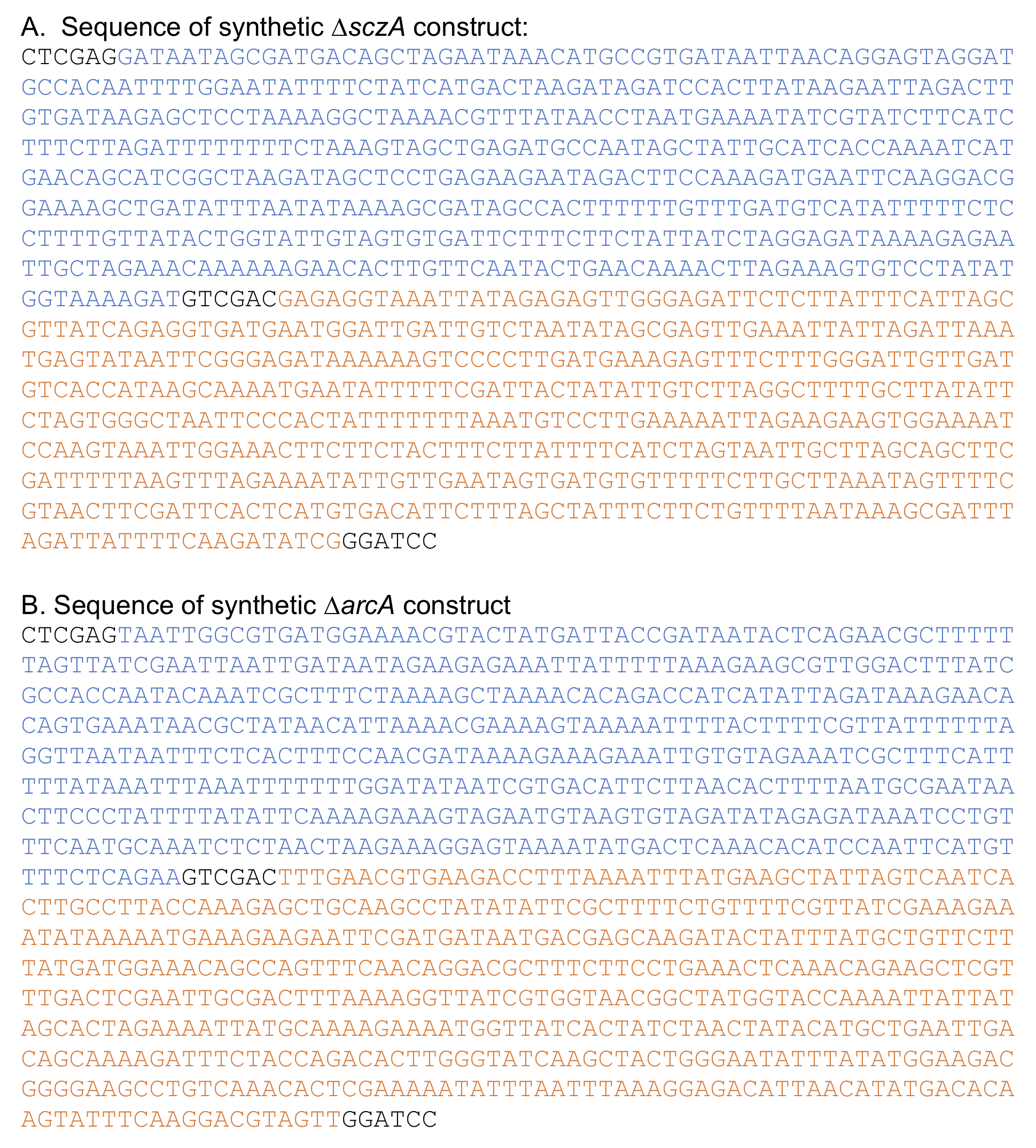

Supplement: FIG S1 [file msphere.00105-21-sf001.tif]

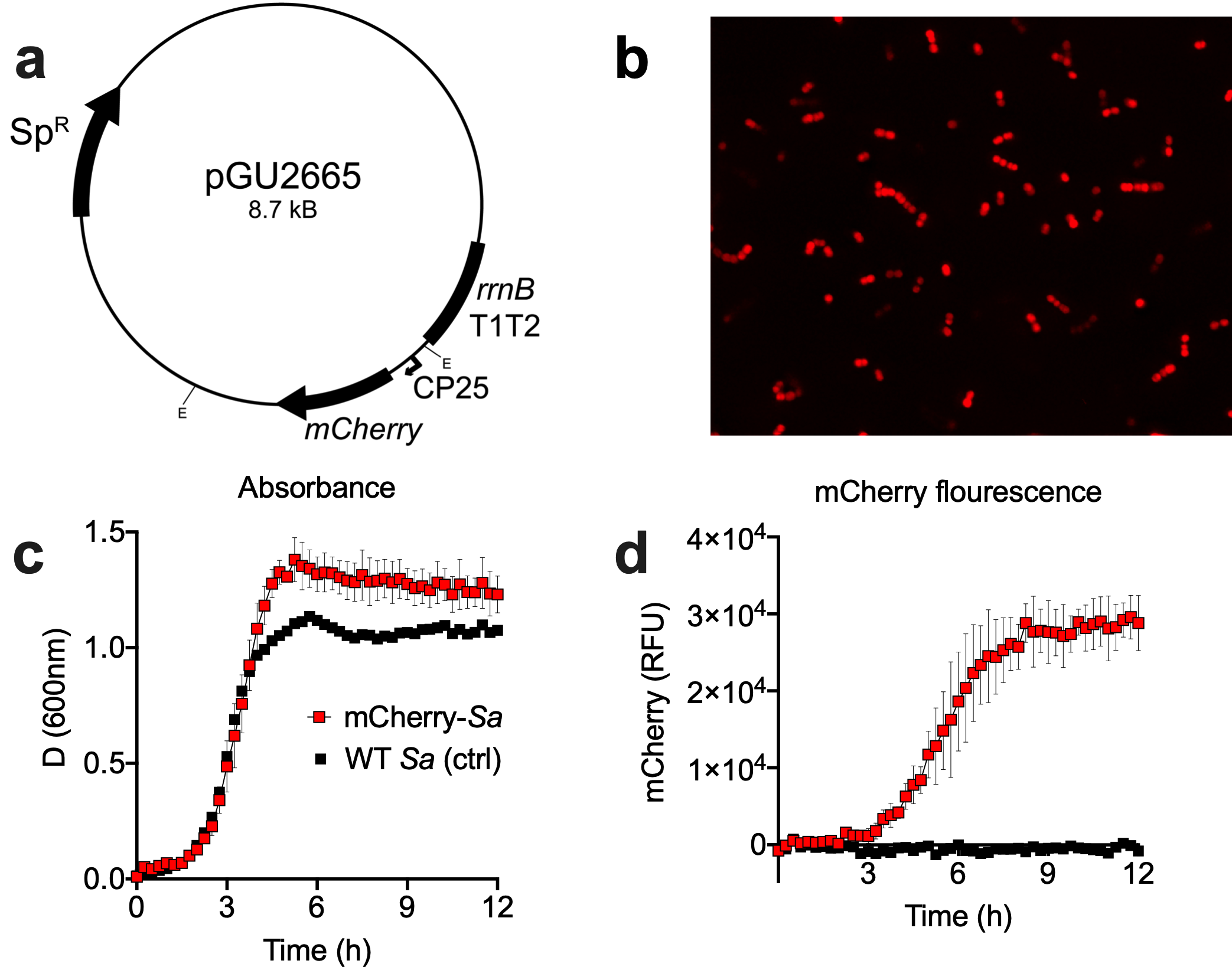

Supplement: FIG S2 [file msphere.00105-21-sf002.tif]
