# Supplementary material for: Cellular Management of Zinc in Group B Streptococcus Supports Bacterial Resistance against Metal Intoxication and Promotes Disseminated Infection
Source: mSphere. 2021 May 19;6(3):e00105-21. doi: 10.1128/mSphere.00105-21 (PMC8265624; doi:10.1128/mSphere.00105-21)
Supplement: TABLE S1 [file msphere.00105-21-st001.docx]

**Supplementary Table 1.** Bacterial strains and plasmids used in this study.

| **Bacteria** | | |
| --- | --- | --- |
| **Strains** | **Characteristics*** | **Source** |
| *E. coli* DH5α | *huA2 lac(*Δ*)U169 phoA glnV44 Φ80' lacZ(*Δ*)M15 gyrA96 recA1 relA1 endA1 thi-1 hsdR17* | Bethesda Research Labs |
| *S. agalactiae* 874391 | Wild type, Sequence type-17, Serotype III strain | (1) |
| *S. agalactiae* GU2487 | 874391Δ*czcD* (*czcD*^-^ mutant); Cm  Locus tag: CHF17_RS02855 | This work |
| *S. agalactiae* GU2791 | 874391Δ*sczA* (*sczA*^-^ mutant)  Locus tag: CHF17_RS02860 | This work |
| *S. agalactiae* GU3042 | 874391Δ*arcA* (*arcA*^-^ mutant)  Locus tag: CHF17_RS11045 | This work |
| *S. agalactiae* GU2667 | Wild type 874391 containing pGU2665 | This work |
| *S. agalactiae* GU2802 | *czcD*-complement (pGU2699) in GU2487, Sp | This work |
| *S. agalactiae* GU2894 | *sczA*-complement (pGU2861) in GU2791, Sp | This work |
| **Plasmids** | | |
| pHY304aad9 | *ori* (Ts); temperature-sensitive shuttle vector; Sp | (2) |
| pLZ12 | *E. coli Streptococcus* shuttle vector; Cm | (3) |
| pMSP3545spec | *E. coli Streptococcus* shuttle vector; Sp | (2) |
| pDL278 | *E. coli Streptococcus* shuttle vector; Sp | (4) |
| pGU2665 | *mCherry::cat* cloned into pDL278; Sp | This work |
| pGU2461 | pHY304*aad9-*derivative *czcD*Δ::*Cm* construct; Cm, Sp | This work |
| pGU2776 | pHY304*aad9-*derivative *sczA*Δ construct; Sp | This work |
| pGU2699 | *czcD* cloned into pMSP3545spec; Sp | This work |
| pGU2861 | *sczA* cloned into pDL278; Sp | This work |
| pGU2777 | pHY304*aad9-*derivative *arcA*Δ construct; Sp | This work |

* Sp = Spectinomycin-resistant; Cm = Chloramphenicol-resistant; Ts = temperature-sensitive

**References**

1. Takahashi S, Nagano Y, Nagano N, Hayashi O, Taguchi F, Okuwaki Y. 1995. Role of C5a-ase in group B streptococcal resistance to opsonophagocytic killing. Infect Immun 63:4764-9.

2. Ipe DS, Ben Zakour NL, Sullivan MJ, Beatson SA, Ulett KB, Benjamin WHJ, Davies MR, Dando SJ, King NP, Cripps AW, Schembri MA, Dougan G, Ulett GC. 2015. Discovery and Characterization of Human-Urine Utilization by Asymptomatic-Bacteriuria-Causing *Streptococcus agalactiae*. Infect Immun 84:307-19.

3. Perez-Casal J, Caparon MG, Scott JR. 1991. Mry, a trans-acting positive regulator of the M protein gene of *Streptococcus pyogenes* with similarity to the receptor proteins of two-component regulatory systems. J Bacteriol 173:2617-24.

4. LeBlanc DJ, Lee LN, Abu-Al-Jaibat A. 1992. Molecular, genetic, and functional analysis of the basic replicon of pVA380-1, a plasmid of oral streptococcal origin. Plasmid 28:130-45.
