# Supplementary material for: Cellular Management of Zinc in Group B Streptococcus Supports Bacterial Resistance against Metal Intoxication and Promotes Disseminated Infection
Source: mSphere. 2021 May 19;6(3):e00105-21. doi: 10.1128/mSphere.00105-21 (PMC8265624; doi:10.1128/mSphere.00105-21)
Supplement: TABLE S2 [file msphere.00105-21-st002.docx]

**Supplementary Table 2.** Oligonucleotides used in this study.

| **Oligos** | | | |
| --- | --- | --- | --- |
| **ID** | **Sequence** | **Usage** |  |
| M13F | GTAAAACGACGGCCAG | Sequencing |  |
| M13R | CAGGAAACAGCTATGAC | Sequencing |  |
| pDL278_seq_F1 | GCCTCTTCGCTATTACGCCA | Sequencing |  |
| pDL278_seq_R1 | CTGGAAAGCGGGCAGTGA | Sequencing |  |
| pMSP3545-MCS-F1 | GCCGATTCATTAATGCAGGT | Sequencing |  |
| pMSP3545-MCS-R1 | TATTGTCGATAACGCGAGCATA | Sequencing |  |
| mCherry_N-term_R1 | GCCTTCACCTTCACCTTCAA | Sequencing |  |
| mCherry_C-term_F1 | GGACGGTGCTTTAAAAGGAGA | Sequencing |  |
| mCherry_C-term_R2 | CATTCCGCCAGTTGAATGAC | Sequencing |  |
| pDL278-Fluoro_F1 | ATTGTCAATATATTCAAGGCAATCTGTCTTGGTCGTCAGACTGATG | Sequencing |  |
| pDL278-Fluoro_F1 | AGGATGAAGAGGATGAGGAGGCGTTCAAAATGGTATGCGTTT | Sequencing |  |
| czcD-Up-F2 | GCACTCGAGAACTAAATACTCTTCAAACG | Cloning |  |
| czcD-Up-R2 | CTAATGTCACTAACCTGCCCCGGAAAAGCTGATATTTAATA | Cloning |  |
| czcD-Down-F1 | CCAATTTTCGTTTGTTGAACTAACATCAAAATCATTGCAAACCA | Cloning |  |
| czcD-Down-R1 | CGATCTGCAGTGAGACACCGATGGATTT | Cloning |  |
| czcD-Chk-F1 | TGTCTTTGATGAAGCCACCA | Sequencing |  |
| czcD-Chk-R1 | TCGAAAGGTGTATTTCCGATG | Sequencing |  |
| czcD-comp-F2 | GAGGATTCAGCATTATAAATCGCTACTTTTGTTTT | Cloning |  |
| czcD-comp-R2 | GAAAGCTTCCACGTACCTAAACCTAAAGTTGG | Cloning |  |
| sczA-chk-F1 | TTCATCATGTAATGCAGCGTAA | Sequencing |  |
| sczA-chk-R1 | TCTTTTGAAAGAAACAGTGGAACA | Sequencing |  |
| arcA-chk-F1 | AAAAAGGTGCACGTTCAAGTA | Sequencing |  |
| arcA-chk-R1 | ACCAAGGTATTCCGGATGAG | Sequencing |  |
| sczA-comp-F1 | GAGAATTCTCATGAACAGCATCGGCTAA | Cloning |  |
| sczA-comp-R1 | GAGTCGACTCACCTCTGATAACGCTAATGAA | Cloning |  |
| dnaN-1F | CAACAAGAAAGCCGTCCAAT | qPCR |  |
| dnaN-1R | TCTGTCGCAACAGCCTTAAA | qPCR |  |
| czcD-1F | TCAATATCTGGTCAATGGATGG | qPCR |  |
| czcD-1R | TAATGTTGGCAAATCGTTCG | qPCR |  |
| sczA-1F | GGAAGTTACCCGATTGAGCA | qPCR |  |
| sczA-1R | TGCCAGGAGGAGAATAGGAA | qPCR |  |
