# Supplementary material for: Cellular Management of Zinc in Group B Streptococcus Supports Bacterial Resistance against Metal Intoxication and Promotes Disseminated Infection
Source: mSphere. 2021 May 19;6(3):e00105-21. doi: 10.1128/mSphere.00105-21 (PMC8265624; doi:10.1128/mSphere.00105-21)
Supplement: TABLE S3 [file msphere.00105-21-st003.docx]

**Supplementary Table 3.** Metal ion concentrations in basal growth media.

| **Metal ion** | **Conc. in THB (μM)** | **Conc. in CDM (μM)** |
| --- | --- | --- |
| Zn | 10.9 (±0.07) | 0.11 (±0.03) |
| Fe | 5.8 (±0.02) | 0.2 (±0.005) |
| Mn | 0.38 (±0.001) | 0.007 (±0.0003) |
| Cu | 0.11 (±0.004) | 0.04 (±0.003) |
